# Characterization of PPE19 as a novel mediator of *Mycobacterium tuberculosis*-macrophage interactions

**DOI:** 10.1128/msphere.00036-25

**Published:** 2025-08-11

**Authors:** Christopher J. De Voss, Sean N. Riek, Miljan Stupar, Lendl Tan, Brian M. Forde, Nicholas P. West

**Affiliations:** 1 School of Chemistry and Molecular Biosciences, The University of Queensland 198110 , Brisbane, Queensland, Australia; 2 UQ Centre for Clinical Research, Faculty of Medicine, The University of Queensland 420004, Brisbane, Queensland, Australia; 3 Institute of Molecular Biosciences, The University of Queensland, Brisbane, Queensland, Australia; 4 Australian Infectious Disease Research Centre, The University of Queensland 1974, Brisbane, Queensland, Australia; Washington University in St. Louis School of Medicine, St. Louis, Missouri, USA

**Keywords:** tuberculosis, PPE/PE proteins, pathogenesis, macrophage, protein secretion

## Abstract

*Mycobacterium tuberculosis* (Mtb) is a highly adapted human pathogen capable of manipulating host immunity. This study demonstrates that PPE19, a member of the PE/PPE protein family, facilitates Mtb adhesion to, and invasion of murine macrophages. PPE19-coated microspheres showed enhanced uptake by macrophages compared to control beads, while Mtb overexpressing *ppe19* (Rv1361c) was phagocytosed at a significantly greater rate than WT Mtb. *ppe19* is identified as pH responsive and displays reduced expression following macrophage entry. CRISPR interference-mediated knockdown of two highly related PPE proteins, *ppe18* (Rv1196) and *ppe60* (Rv3478), revealed an additive reduction in Mtb’s ability to invade host macrophages, indicating a potential functional relationship. Furthermore, the absence of an *in vivo* phenotype following murine infection with a *ppe19* knockout strain suggests functional redundancy within this PPE protein family. Finally, PE13 has been identified here as a binding partner for PPE19, characterizing another relationship presumed important for successful PPE secretion. These findings reveal PPE19 as a secreted effector protein used by Mtb to modulate important early interactions with the innate immune system, enhancing entry into host macrophages.

**IMPORTANCE:**

Tuberculosis remains a leading infectious disease killer worldwide, with approximately one-quarter of the global population infected with *Mycobacterium tuberculosis* (Mtb). Understanding how this pathogen initially establishes infection is crucial for developing more effective vaccines and treatments. This study identifies PPE19, a previously uncharacterized bacterial protein, as a key factor that helps Mtb invade and colonize human immune cells called macrophages during the earliest stages of infection. The research shows that PPE19 acts like a molecular “key” that facilitates bacterial entry into host cells but is then downregulated once the bacteria are safely inside. Importantly, PPE19 belongs to a family of similar proteins that can compensate for each other, explaining why targeting individual members may not be sufficient for treatment. These findings provide new insights into tuberculosis pathogenesis and suggest that early infection factors like PPE19 could serve as targets for next-generation vaccines designed to prevent initial infection rather than just disease progression.

## INTRODUCTION

Tuberculosis (TB), caused by the highly adapted human pathogen *Mycobacterium tuberculosis* (Mtb), is an infectious disease of enormous global significance. Currently, approximately one-quarter of the world’s population is infected with Mtb (1). Of the 10.6 million estimated new TB cases in 2022, 1.3 million people died from the disease worldwide (1), highlighting the devastating global burden of the disease. Mtb is an intracellular pathogen adept at non-replicative persistence during latent stages of disease (2–4), thus presenting many challenges to effective therapy and vaccine development. The pathogenic processes employed by Mtb for metabolic tuning and immune evasion remain poorly understood, and mechanisms of bacterial-host cell communication and manipulation require urgent definition.

Approximately 7% of the coding capacity in the Mtb genome is devoted to the *pe/ppe* gene family (5), indicating its significant role in bacterial pathogenic success. These genes encode glycine-rich proteins, characterized by highly conserved motifs of N-terminal proline-glutamate (PE) or proline-proline-glutamate (PPE). Of the 99 *pe* and 69 *ppe* genes annotated in the laboratory strain Mtb H37Rv (6), the vast majority are absent from avirulent mycobacteria (7), indicating important pathogenic functions of these proteins. Of the PE/PPE proteins with known functions, many contribute vital roles during Mtb infection, including inhibition of oxidative defenses or essential nutrient uptake for the pathogen (8–14). Moreover, through PE/PPE-mediated processes, Mtb modulates pro- (15, 16) and anti-inflammatory (17, 18) host responses, promoting either necrosis (19, 20) or pyroptosis (21) when favorable and restricting antigen presentation by inhibiting autophagy in infected host cells (22–24).

To facilitate the secretion of PPE proteins, Mtb possesses five Type VII secretion systems (T7SS), named ESAT-6-like secretion systems 1–5 (ESX1-5) (25). It has been demonstrated that numerous PPE proteins form a heterodimeric complex with a cognate PE partner protein (26, 27), crucial for secretion through the Type VII systems. Conserved motifs within the PE and PPE proteins are juxtaposed to form a composite secretion sequence specific for the T7SS (28). Given the reliance on PE partners for secretion, and thus the facilitating biological function of a respective PPE, it is important to identify any PE partners for PPE proteins of interest. Genomic interrogation of the laboratory strain H37Rv identified 49 orphaned *ppe* genes transcriptionally unlinked to a *pe* gene. One such orphaned gene, *ppe19* (Rv1361c), clusters separately into a clade together with *ppe18* (Rv1196) and *ppe60* (Rv3478). Interestingly, this clade represents the three members of the protein family with the highest level of similarity among all PPE proteins. This cluster is of particular interest due to the immunological importance of its members (PPE18 and PPE60), including as part of effective candidate vaccines against TB.

PPE18 is one of the two antigens in the candidate subunit vaccine, M72/AS01_E_, which commenced Phase 3 clinical trials in 2024 after showing 54% protection against active pulmonary TB in a Phase 2b trial (29). PPE18 is required by Mtb to establish an effective infection *in vivo* (30) and has been identified as an interacting antigen of Toll-like receptor 2 (TLR-2) to inhibit pro-inflammatory immune responses (31). Furthermore, PPE18 may even impair MHC class-II antigen presentation *in vivo* (32). Like PPE18, PPE60 appears to stimulate TLR-2 and has been proposed as a novel TB vaccine antigen due to its induction of strong T helper (T_H_)1 and T_H_17 immune responses (33). Despite these proteins being of significant interest to TB and vaccine research, the pathogenic roles of the protein cluster still require elucidation. Of the three proteins in the cluster, PPE19 remains the least characterized and has not previously been associated with a known interacting PE partner, and as such, its putative secretion capacity remains in question.

In this study, we conducted an initial characterization of PPE19 to assign pathogenic importance and identify any secretion-essential relationships. We demonstrated that PPE19 plays a significant role during the preliminary stages of infection, significantly enhancing Mtb attachment and internalization into macrophages. The effect of overexpression or genetic knockout of *ppe19* on virulence was investigated in a murine model, with results suggesting that the bacterium possesses alternate effector proteins by which it compensates for *ppe19* expression changes. Finally, we identified PE13 as capable of interacting with PPE19, presumably for its effective secretion and Mtb cell wall localization. These results establish a pathogenic role for PPE19 in manipulating macrophage interactions, and murine *in vivo* infection studies suggest functional redundancy within the cluster of similar PPE proteins. The data presented here further contribute to the increasingly significant evidence supporting PPE proteins as virulence effectors and contributors of pathogenesis.

## RESULTS

### Phylogenetic organization of PPE proteins in Mtb

**Fig 1:**
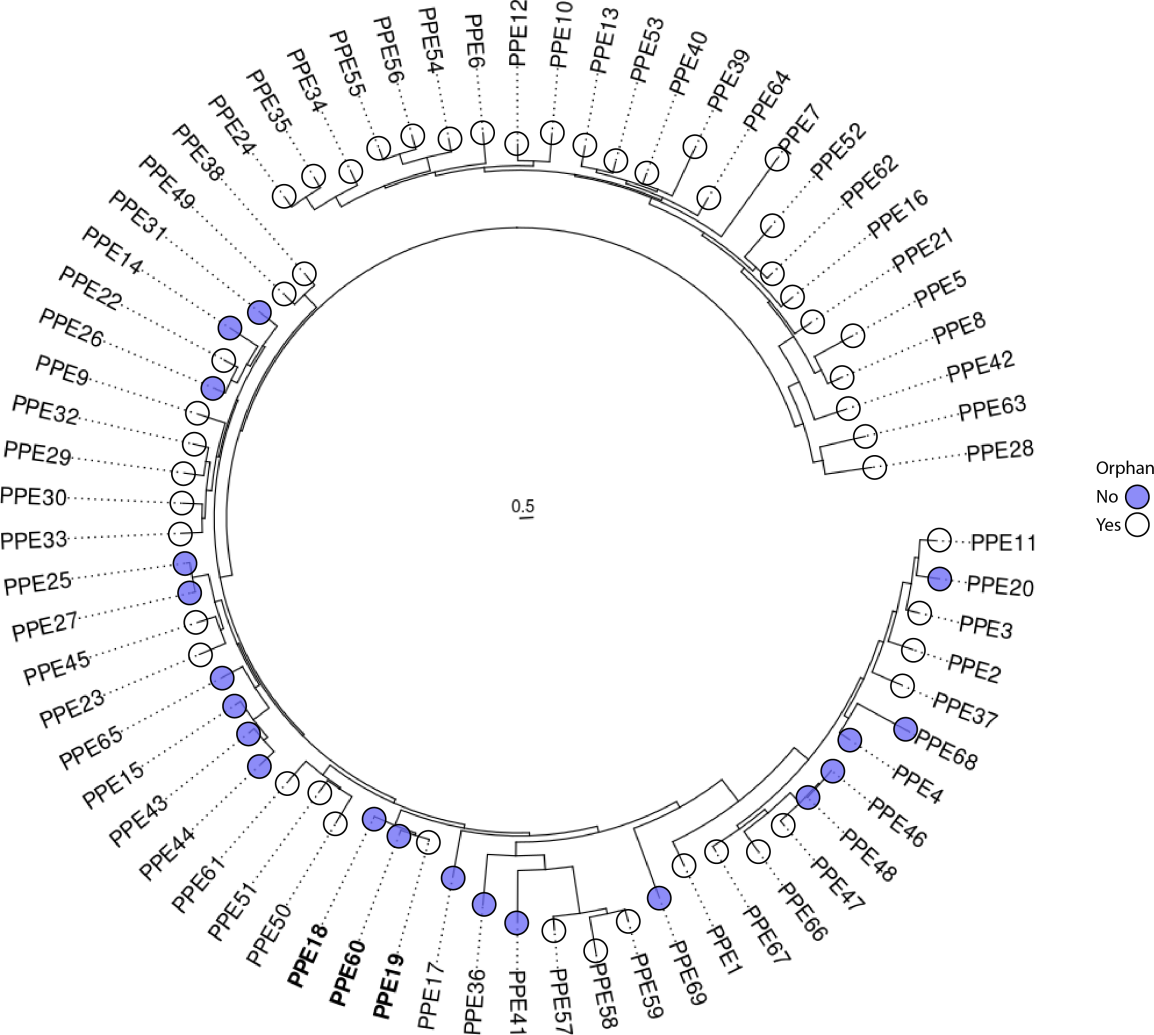
Phylogenetic analysis of the PPE family of proteins. Maximum likelihood phylogenetic tree reconstructed from the alignment of 69 PPE amino acid sequences from the genome of *M. tuberculosis* H37Rv. The scale bar indicates evolutionary distance in amino acid substitutions per site. PPE19 falls into a distinct and closely related evolutionary clade with PPE18 and PPE60 (bold). Blue represents *ppe* genes with a genomically linked *pe* partner. Open circles represent transcriptionally orphaned *ppe* genes.

Phylogeny mapping of the PPE protein family of Mtb reveals a distinct and exceptionally close evolutionary relationship between PPE18, PPE19, and PPE60 (Fig. 1). Of the three proteins, PPE19 is the only orphaned PPE, lacking a genomically associated PE secretion partner. Previous investigations have found PPE18 and PPE60 to play important immunological roles during Mtb infection. PPE18 is required by Mtb to establish an effective infection *in vivo* (30), impairing MHC-II antigen presentation (32) and interacting with TLR-2 to inhibit pro-inflammatory responses (31). It is also one of two antigenic components in the M72F/AS01_E_ subunit vaccine (29). Similarly, PPE60 stimulates TLR-2 and induces robust T_H_1 and T_H_17 responses (32). As PPE19 not only forms a distinct evolutionary clade with PPE18 and PPE60 but also shares a remarkable level of similarity to these PPEs (Fig. 1), we sought to understand the contribution of PPE19 to Mtb infection.

### PPE19 contributes to macrophage interactions and uptake of Mtb

**Fig 2:**
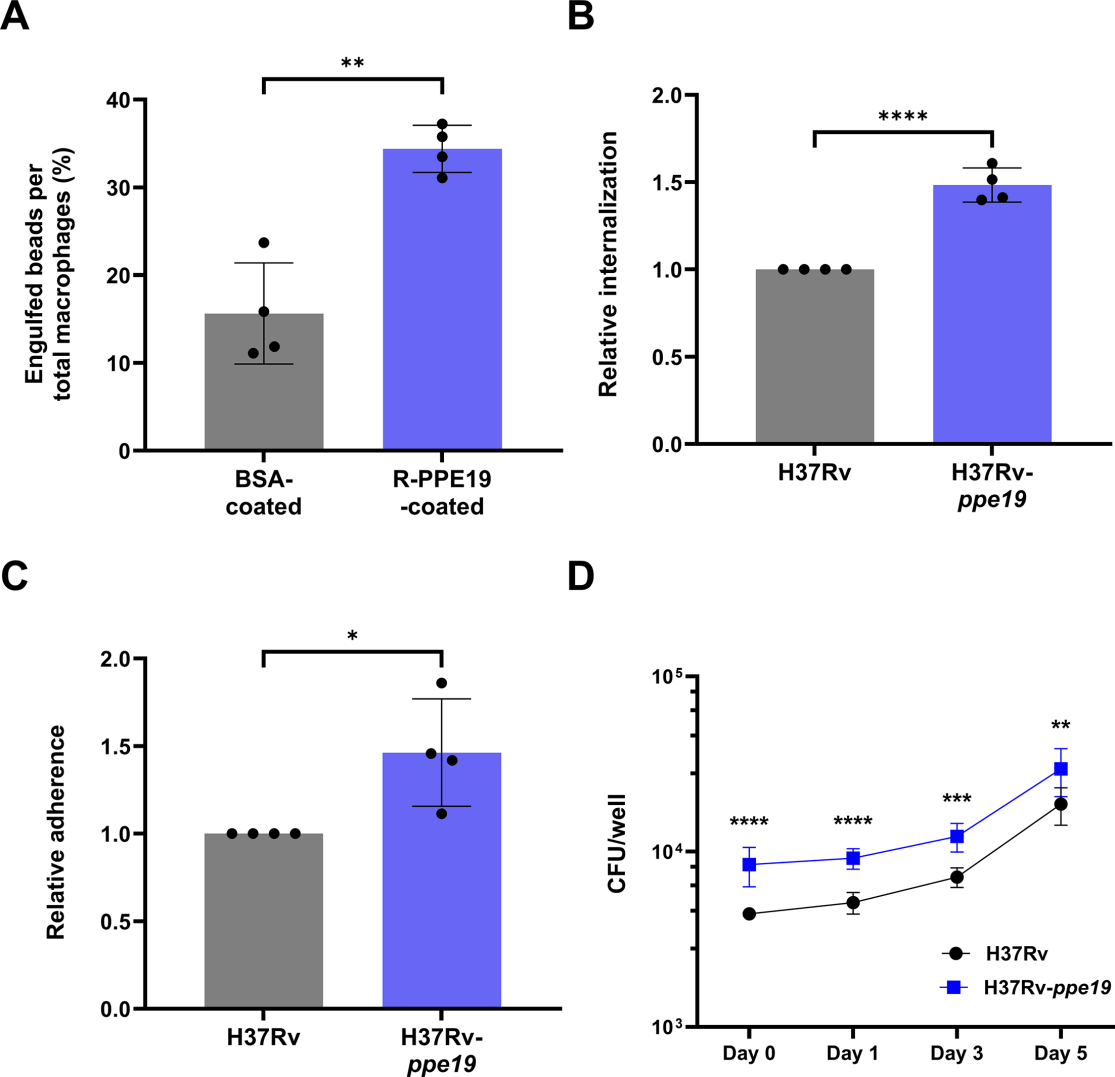
PPE19 enhances phagocytic uptake by macrophages. (**A**) Quantification of BSA- or PPE19-coated FluoSpheres attached or internalized into RAW264.7 macrophages following 4 hours of incubation at an MOI of 4. Data are expressed as a percentage of beads per 1,000 imaged macrophages in each condition, and each point indicates counts from quadruplicate micrographs. The graph is representative of two biological replicates. (**B**) Internalization or (**C**) adherence of H37Rv overexpressing *ppe19* (H37RV-*ppe19*), relative to H37Rv, following infection of THP-1 macrophages for 4 hours at an MOI of 10. Strains were normalized to inocula before relative computation. Data points indicate biological replicates, each representative of six technical replicates. (**D**) Burden of H37Rv or H37Rv-*ppe19* in THP-1 macrophages over a 5-day infection at an MOI of 1. The graph is representative of three biological replicates, and data points indicate six technical replicates. Data are expressed as mean with SD. Significance was calculated using unpaired *t*-tests (**A–C**) or two-way analysis of variance (with Sidak’s multiple comparisons test) (**D**). *P < 0.05; **P < 0.01; ***P < 0.001; and ****P < 0.0001.

Given the principal role of macrophages in Mtb pathogenesis, we examined interactions between this cellular niche and PPE19. To assess the phenotypic response to PPE19, murine RAW264.7 macrophages were incubated with fluorescent microspheres coated with endotoxin-free PPE19 or BSA as an inert control. Confocal imaging of macrophages revealed a greater than twofold increase in the number of engulfed microspheres coated in PPE19 compared to control beads (Fig. 2A; Fig. S1A). The purified PPE19 used in this experiment (Fig. S1B) was treated for endotoxin removal and validated (Fig. S1C) using a transfected RAW264.7 cell-based assay testing for TLR activation (34) to ensure that increased uptake was not due to LPS contamination of PPE19 during recombinant protein purification from *Escherichia coli*. To explore the effect of PPE19 on phagocytic uptake at the cellular level, we constructed a constitutive PPE19 overexpressing strain of Mtb (H37Rv-*ppe19*). The human macrophage-like cell line THP-1 was infected with H37Rv or H37Rv-*ppe19*. Increased macrophage internalization (Fig. 2B) and adhesion (Fig. 2C) were observed following 4 hours of infection at an MOI of 1 with H37Rv-*ppe19* compared to H37Rv. During these early stages of infection, H37Rv-*ppe19* was consistently internalized approximately 1.5-fold more than H37Rv (normalized to inoculum). Macrophage adherence of the PPE19-overexpressing strain was detected at similarly increased proportions. Intracellular bacterial loads were assessed over 5 days to investigate the effect of PPE19 during a longer-term infection. H37Rv-ppe19 was internalized at numbers almost twofold higher compared to H37Rv, but this did not lead to an increase in bacterial replication rate once inside macrophages (Fig. 2D; Fig. S2). When comparing the ratio of intracellular bacteria for each strain at day 3:day 0 (D3:D0) and day 5:day 0 (D5:D0), we observed a slight decrease in the ppe19 over-expressing strain. The D3:D0 ratios were 1.62 for H37Rv and 1.45 for H37Rv-ppe19, while the D5:D0 ratios were 4.22 for H37Rv compared to 3.51 for H37Rv-ppe19. We hypothesize that the fitness cost associated with the constitutive overexpression of *ppe19* may contribute to this reduction in intracellular replication rate. While the overexpression of *ppe19* contributed to the initial uptake of Mtb H37Rv into macrophages, it does not facilitate increased bacterial replication once internalized. Taken together, these data demonstrate that PPE19 enhances Mtb adhesion to and uptake by host macrophages, contributing to the initial stages of pathogenesis and promoting efficient cellular colonization.

### Downregulation of *ppe19* expression upon internalization into macrophages

**Fig 3:**
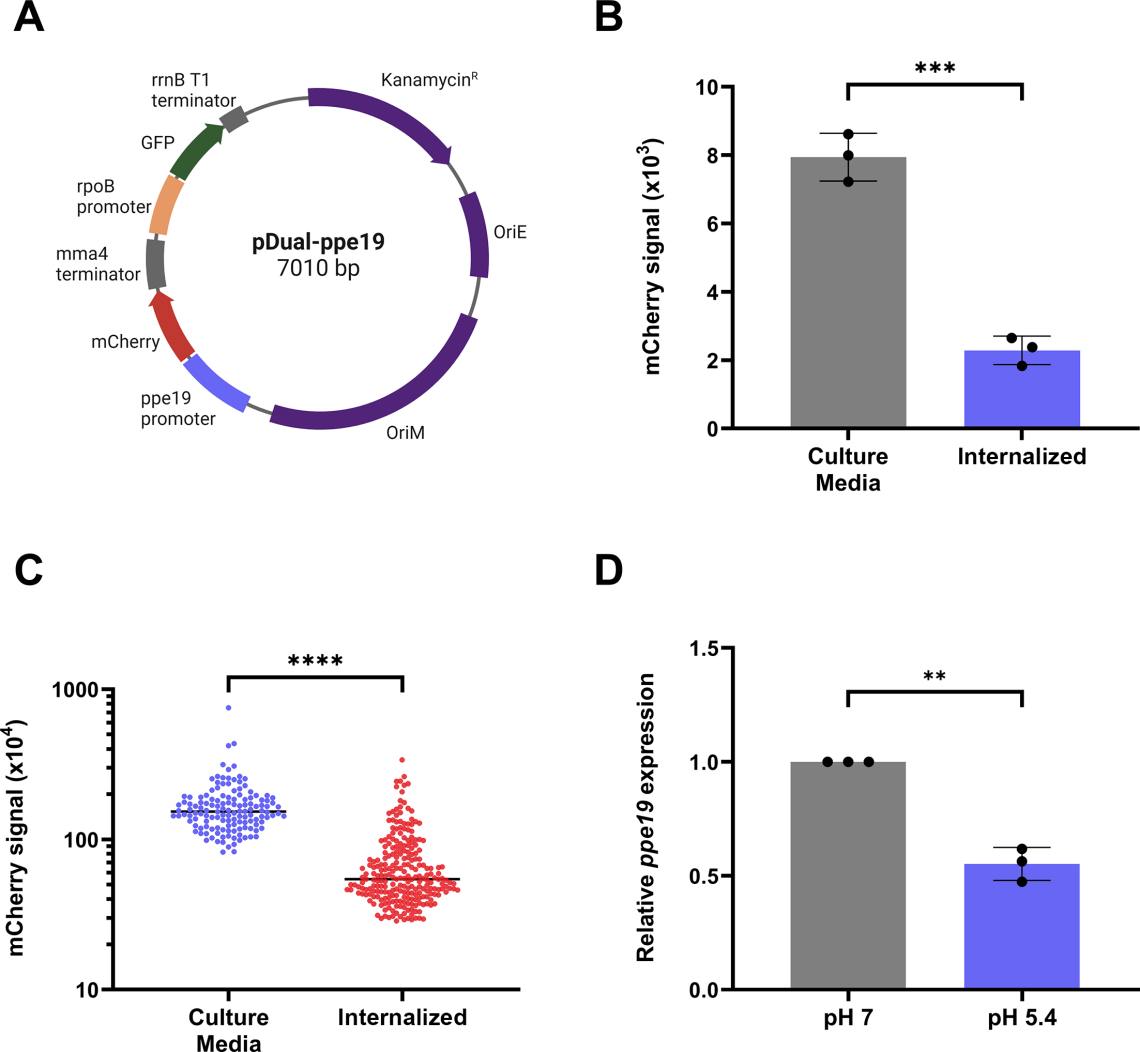
Expression of *ppe19* is downregulated following Mtb infection of macrophages. (**A**) Map of the pDual-*ppe19* vector, encoding *mCherry* and *gfp* reporter genes transcriptionally coupled to the putative *ppe19* promoter region and the *rpoB* promoter, respectively. (**B**) Level of mCherry fluorescence was measured in H37Rv::pDual-P*ppe19* grown in 7H9 media or following infection of THP-1 macrophages for 4 hours at an MOI of 5. Analysis was conducted by flow cytometry, with the bacterial population identified by GFP signal. (**C**) Level of mCherry fluorescence in H37Rv::pDua*l-Pppe19* quantified using confocal microscopy and analyzed in Imaris, following the same experimental protocol as panel **B**. (**D**) Relative ppe19 gene expression in H37Rv in standard pH 7 7H9 media or 7H9 buffered to pH 5.4, measured by qRT-PCR. Data points in panels **B** and **D** indicate biological replicates representative of three technical replicates. Data points in panel **C** represent fluorescence from individual bacilli. Data in panel **B** are expressed as median with SD, and data in panels **C** and **D** are expressed as mean with SD. Significance was calculated using unpaired *t*-tests. *P < 0.05; **P < 0.01; ***P < 0.001; and ****P < 0.0001.

Having repeatedly observed the stimulatory effect of PPE19 on phagocytic uptake, it was important to correlate this potential virulence function with the expression profile of *ppe19* during Mtb infection. We therefore created a transcriptional reporter vector, pDual-P*ppe19* (Fig. 3A), containing the 554 bp region immediately upstream of *ppe19*, which is proposed to include the putative *ppe19* promoter region. This upstream region was transcriptionally coupled to the fluorescent protein, *mCherry*, while a second fluorophore, GFP, was placed under the control of the constitutive *rpoB* promoter as an internal control of basal Mtb gene expression, together with bacterial cell normalization (Fig. 3A). Macrophages were infected with Mtb containing this vector (H37Rv::pDual-Pppe19), with mCherry fluorescence subsequently quantified within this population, normalized to GFP fluorescence. Following infection of THP-1 macrophages for 4 hours at an MOI of 5, we observed an approximate fourfold reduction in mCherry fluorescence in bacteria internalized or adhered to macrophages compared to bacteria grown in liquid culture (Fig. 3B). This analysis was conducted using flow cytometry, with bacterial populations identified by GFP signal (Fig. 3B). The observed reduction in *ppe19* promoter activity was corroborated through visualization of identical replicate infections by confocal microscopy and subsequent quantification of mCherry signal of hundreds of individual bacilli using Imaris (Fig S3 for representative micrographs). Attached or internalized H37Rv::pDual-P*ppe19* within THP-1 cells had a significant 2.4-fold reduction in mCherry signal compared to those grown in liquid culture using this quantification method (Fig. 3C). As an independent method of investigating *ppe19* transcriptional activity following phagocytic uptake of Mtb, qRT-PCR was used to directly measure *ppe19* gene expression by Mtb during acid stress. Acidification of phagosomes is a well-defined defense mechanism employed by macrophages against Mtb (35, 36) and a key environmental signal to the pathogen of phagocytic uptake (37–39). Indeed, qRT-PCR revealed a 1.8-fold downregulation of *ppe19* gene expression by Mtb H37Rv exposed to acid stress for 24 hours compared to bacteria in standard pH 7 media (Fig. 3D). These findings suggest that PPE19 is involved in the initial invasion of Mtb into host macrophages, and once internalized, *ppe19* expression is downregulated as the pathogen no longer requires factors to enhance cellular entry but rather prepares to establish immunological persistence.

### Overexpression of PPE19 *in vivo* does not increase the virulence of Mtb

**Fig 4:**
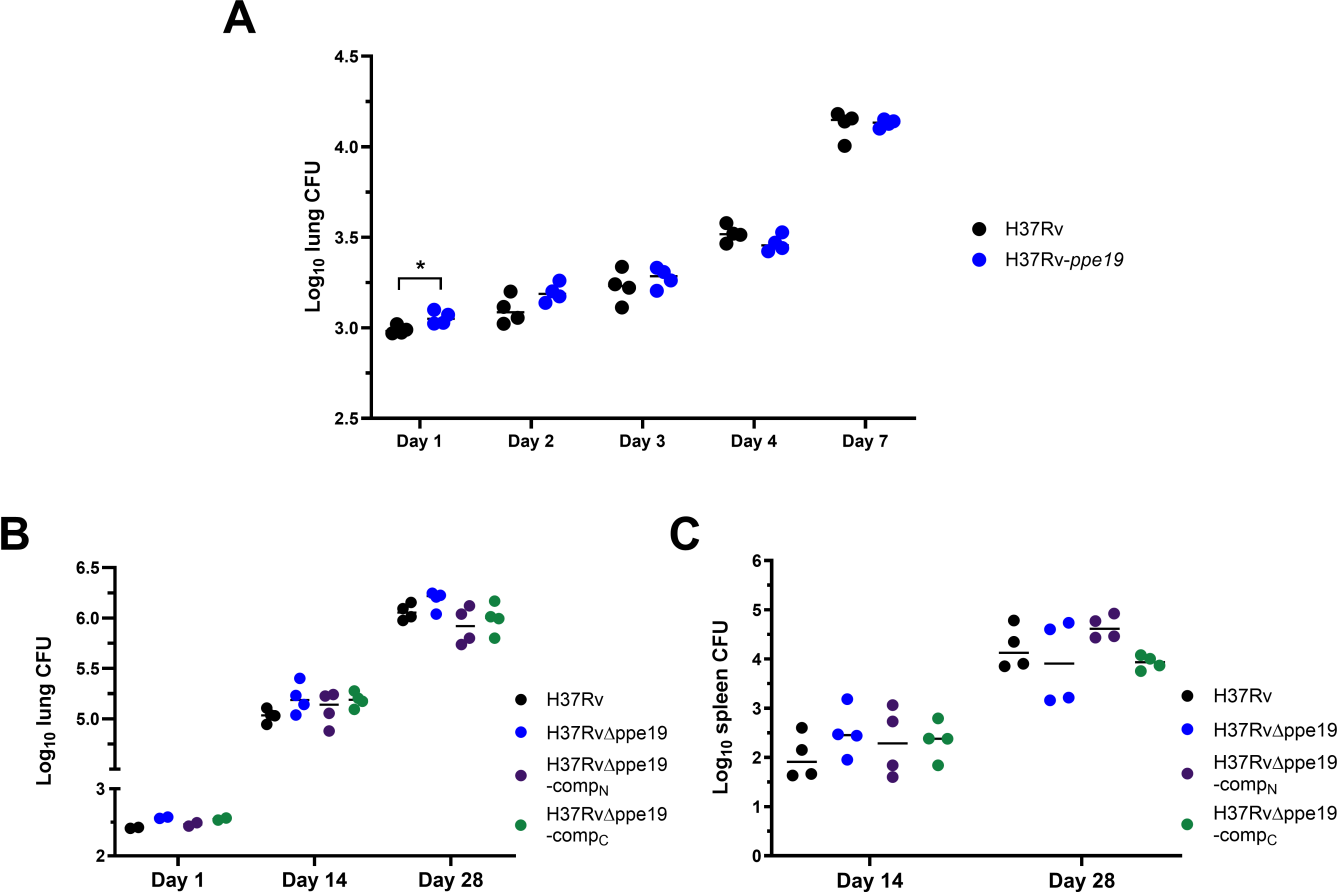
Increased or abolished *ppe19* expression does not affect *in vivo* virulence of Mtb. (**A**) Mtb burden in the lungs of C57BL/6 mice infected with H37Rv or H37Rv overexpressing *ppe19* (H37Rv-*ppe19*) over 7 days of infection. Data are CFU counts from lung homogenates of four individual animals per group per time point. (**B**) Mtb burden in the lungs or (**C**) spleens of C57BL/6 mice infected with H37Rv, a *ppe19* knockout mutant (H37RvΔ*ppe19*), or two complemented strains with *ppe19* expression restored on a native (H37RvΔ*ppe19*-comp_N_) or constitutive (H37RvΔ*ppe19*-comp_C_) promoter. Data are CFU counts from organ homogenates of four individual animals per group time point. Horizontal line indicates the mean. Significance in panel **A** was calculated using multiple, unpaired, two-tailed *t*-tests. Significance in panels **B** and **C** was calculated using one-way analysis of variance with Tukey’s multiple comparisons test. *P < 0.05.

To investigate whether the *in vitro* ability of H37Rv-*ppe19* to enhance macrophage phagocytic uptake would alter the overall virulence of Mtb *in vivo*, C57BL/6 mice were challenged with an aerosol infection of ~10^3^ CFU of H37Rv or H37Rv-*ppe19*. The bacterial burden in the lungs of these mice was assessed over a short infection timeframe of 7 days to capture any initial or immediate impacts of altered *ppe19* expression. While the mean CFU in the lungs of H37Rv-*ppe19-*infected mice was greater than H37Rv-infected animals at days 1, 2, 3, and 7, this difference failed to reach statistical significance aside from day 1 (Fig. 4A). To further probe the role of PPE19 *in vivo*, a *ppe19* knockout in Mtb was created (MtbΔ*ppe19*). This strain was used in an aerosol infection of C57BL/6 mice, alongside H37Rv and two complemented strains, with *ppe19* expression restored under its native (H37RvΔ*ppe19*-comp_n_) or a constitutive (H37RvΔ*ppe19*-comp_c_) promoter. All groups received ~2–3 × 10^2^ CFU of the relevant strain, as assessed through lung CFU of two mice per group at 1 day post-infection. No significant differences in Mtb burden were observed in the lung or spleens of mice in any groups at 14- or 28-days post-infection, except between the knockout and native complement strains at day 28 in the lung (Fig. 4B and C). With no obvious *in vivo* phenotype observed, together with the extremely high sequence homology between *ppe18*, *ppe19*, and *ppe60*, we propose that *ppe18* and *ppe60* may compensate for the loss of *ppe19 in vivo*.

### Functional redundancy for PPE19 may be afforded by closely related PPE proteins

**Fig 5:**
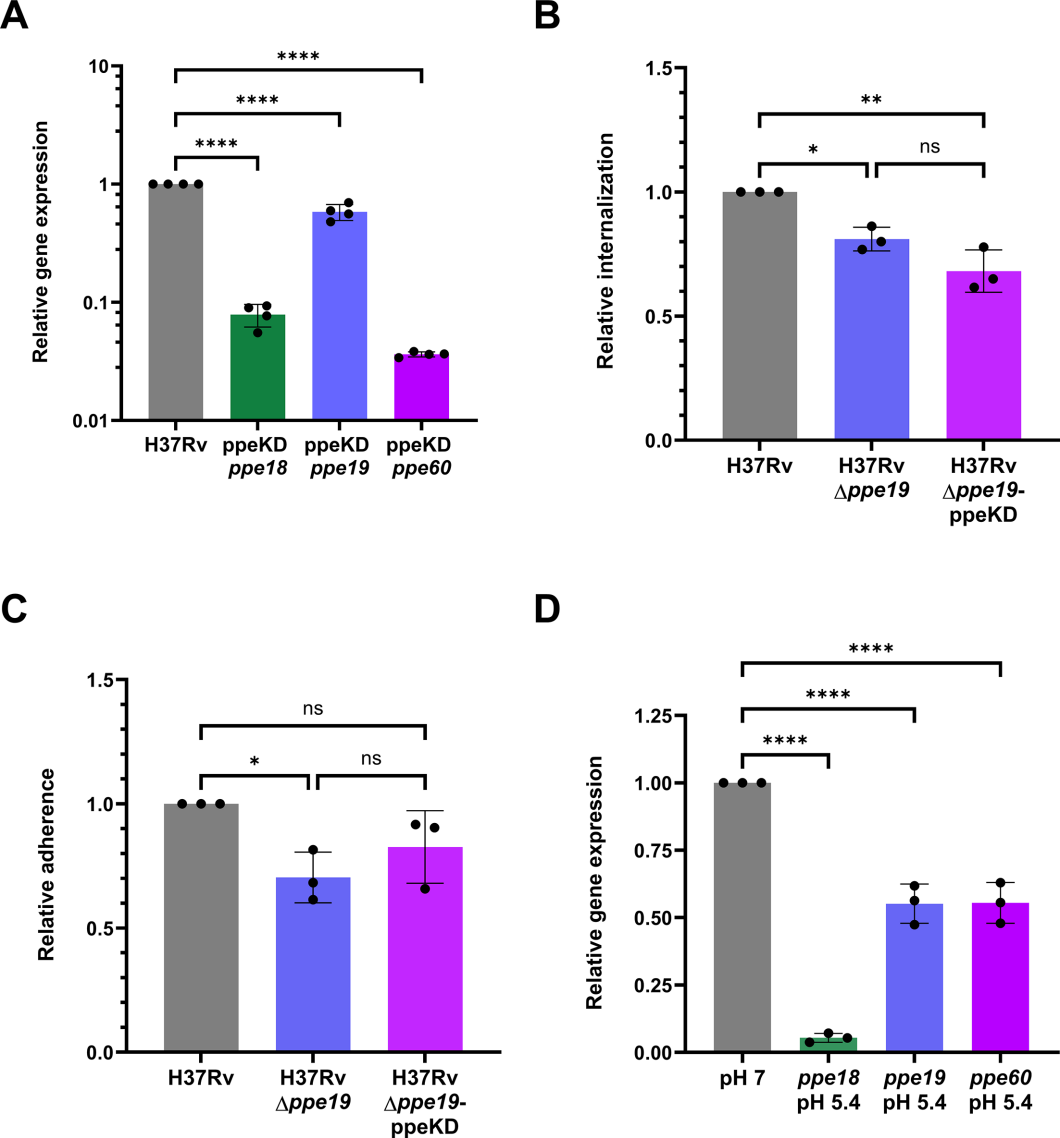
Related genes *ppe18* and *ppe60* may provide some functional redundancy for *ppe19*. (**A**) Relative expression of *ppe18*, *ppe19*, and *ppe60* following simultaneous transcriptional repression of all three genes in H37Rv through CRISPRi. Data points indicate technical replicates from RT-qPCR, and the graph is representative of duplicate experiments. (**B**) Internalization or (**C**) adherence of a *ppe19* knockout mutant (H37RvΔ*ppe19*) or H37RvΔ*ppe19* containing an integrative CRISPRi vector to simultaneously repress the transcription of *ppe18* and *ppe60* (H37RvΔ*ppe19*-ppeKD), relative to H37Rv, following 4 hours of infection of THP-1 macrophages at an MOI of 10. Each strain was normalized to the original inoculum before relative computation. Data points indicate biological replicates, each representative of four technical replicates. (**D**) Relative expression of *ppe18*, *ppe19,* and *ppe60* at pH 5.4, relative to each gene’s expression at pH 7. Data points indicate biological replicates from RT-qPCR, each representative of technical replicates. Data are expressed as mean with SD. Significance was calculated using one-way analysis of variance with Tukey’s multiple comparisons test. ns, not significant; *P < 0.05; **P < 0.01; and ****P < 0.0001.

Mtb possesses a variety of virulence factors enabling invasion into host cells (40–43), indicating redundancy acquired through millennia of host-pathogen coevolution (44). To examine if *ppe18* and *ppe60* also influence phagocytic uptake, two small guide RNAs (sgRNAs) were designed for CRISPRi-based transcriptional repression as described (45) to simultaneously target *ppe18*, *ppe19*, and *ppe60*. RT-qPCR revealed that both sgRNAs were equally efficacious at transcriptional repression of the target genes, with the selected sgRNA construct achieving a 12.7-fold reduction in *ppe18* expression, a 1.7-fold reduction in *ppe19* expression, and a 27.6-fold reduction in *ppe60* expression following CRISPRi induction in H37Rv (Fig. 5A; Fig. S4). To investigate any functional redundancies within this *ppe* trio related to phagocytic uptake, this vector was transformed into the *ppe* knockout strain, H37RvΔ*ppe19,* to generate H37RvΔ*ppe19*-ppeKD. THP-1 cells were infected with H37Rv, H37RvΔ*ppe19*, or H37RvΔ*ppe19*-ppeKD. Reduced macrophage internalization (Fig. 5B) was observed for both H37RvΔ*ppe19* and H37RvΔ*ppe19*-ppeKD compared to the wild-type control following 4 hours of infection at an MOI of 10. While this reduction was more pronounced in the tri-*ppe* knockdown strain, there was no statistical difference between this and the *ppe19* knockout alone. Both strains had a reduced adherence to macrophages compared to H37Rv, but only the *ppe19* knockout strain reached statistical significance (Fig. 5C). However, as reported here, the measured adherence values of all strains were more variable than internalization values. To investigate similarities in gene expression regulation, the relative expression of each *ppe* was analyzed using RT-qPCR. Each *ppe* was downregulated following Mtb exposure to acid stress of 5.4 for 24 hours (Fig. 5D), compared to expression at pH 7. We observed an almost identical downregulation of *ppe19* and *ppe60* by 1.8-fold. Strikingly, *ppe18* was downregulated 18.4-fold, indicating the high receptiveness of *ppe18* modulation due to acid stress (Fig. 5D). Together, these data indicate that there may be some functional redundancy related to phagocytic uptake afforded by *ppe18* and *ppe60*. However, within this trio of *ppes*, *ppe19* appears to be the key mediator of Mtb macrophage uptake *in vitro*.

### Identification of a potential PE secretion partner for PPE19

**Fig 6:**
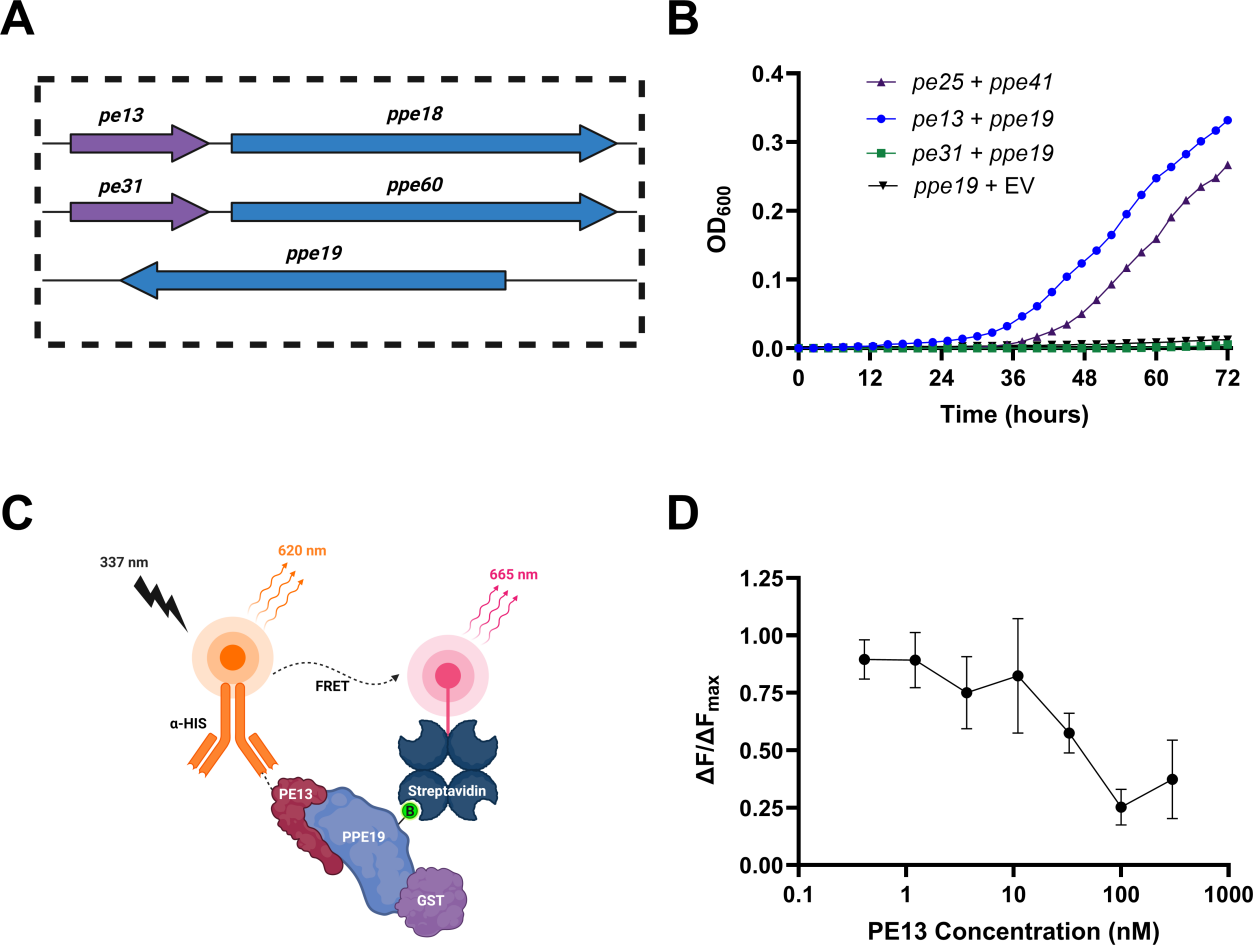
PE13 is a binding partner of PPE19. (**A**) Genomic organization of *ppe18*, *ppe19*, and *ppe60* in Mtb H37Rv, with present adjacent *pe* genes indicated. (**B**) A mycobacterial protein fragment complementation assay, showing OD_600_ growth of Ms strains in 6.25 µg/mL trimethoprim. Legend of “*pe + ppe”* indicates relevant genes on separate vectors in each strain, with empty vector (EV) control indicated. Data points indicate three technical replicates, and the graph is representative of four biological replicates. (**C**) Schematic of homogeneous time resolved fluorescence (HTRF) assay performed here. PE13-His is recognized by the HTRF donor (terbium cryptate-conjugated anti-6-His antibody), while PPE19-GST is biotinylated, allowing binding to HTRF acceptor (streptavidin-d2). Excitation of terbium cryptate at 340 nm causes emission at 665 nm, but if the donor and acceptor are in close enough proximity, d2 emission will occur at 620 nm. (**D**) Relative HTRF signals across a concentration gradient of PE13-His for interaction with 33 nM biotinylated PPE19-GST (PPE19-GST-bio). Data points are a combination of three replicate experiments. Data are expressed as mean with SD.

Given the role of a partner PE protein in facilitating PPE secretion, we were interested in whether an interacting PE partner protein could be identified for effective PPE characterization and functional studies. Since there is no *pe* adjacent to *ppe19* in the Mtb genome, we relied on our phylogenetic analysis of the 69 *ppe* genes of Mtb H37Rv to identify closely related *ppes* (Fig. 1). We predicted that if a highly similar *ppe* to *ppe19* has a partner *pe*, then that PE protein may be capable of interacting with PPE19. This analysis revealed a distinct evolutionary clade containing *ppe18*, *ppe19*, and *ppe60* (Fig. 1), reinforced by the >90% base pair similarity across the three genes identified by multiple sequence alignment. Given that *ppe18* and *ppe60* are located immediately downstream of *pe13* and *pe31,* respectively (Fig. 6A), these two PE proteins were selected as potential candidates to interact with PPE19. This prediction aligns with another made previously by a distinct computational means (46). We used a mycobacterial two-hybrid assay system as previously described (47) to provide a more “native” intracellular environment for mycobacterial protein studies in *Mycobacterium smegmatis* (Ms). In this mycobacterial protein fragment complementation assay, PPE19 was expressed at the N-terminus of one fragment of murine dihydrofolate reductase (mDHFR), while PE13 or PE31 was expressed as fusion proteins with the second mDHFR fragment to identify PE/PPE interactions by Ms resistance to the antibiotic trimethoprim as conferred through formation of the mDHFR holoenzyme. A clear interaction was observed between PE13 and PPE19, even greater than that displayed by the known interacting PE/PPE pair, PE25/PPE41 (Fig. 6B). Conversely, the PE31/PPE19 strain yielded no trimethoprim resistance (Fig. 6B).

To validate PE13/PPE19 interaction, both proteins were expressed recombinantly in *Escherichia coli*, following a chaperone-assisted protocol for the expression of soluble PE/PPE proteins devised here and using an *E. coli* codon-optimized sequence of *ppe19*. PPE19 was purified with an N-terminal GST tag and subsequently biotinylated to produce PPE19-GST-bio (Fig. S5A), while PE13 was purified with a polyhistidine affinity tag to isolate PE13-His (Fig. S5B). We utilized homogeneous time resolved fluorescence (HTRF) as a proximity assay to identify protein interaction, as illustrated by the cartoon representation (Fig. 6C). A single concentration of 33 nM of PPE19-GST-bio was used in this assay due to its limited stability. Numerous HTRF assays revealed a replicable and specific signal for interaction between PE13 and PPE19, with the strongest interaction signals consistently observed at PE13 concentrations of 0.41 and 1.67 nM (Fig. 6D). While other PE partners for PPE19 may be present in the Mtb genome, these data indicate that PE13 is a capable interacting partner of PPE19.

## DISCUSSION

The data presented in this study support the view that PPE19 is an effector protein of Mtb and contributes to its virulence, most likely by acting as a secreted cell-surface adhesion factor and facilitating macrophage uptake of Mtb. Uptake of Mtb into macrophages is a critical first step in the infectious lifecycle of the pathogen, allowing Mtb access to its intracellular niche and for the bacteria to establish infection (3, 40, 48). The ability of Mtb to evade innate immunity is emphasized by its incredibly low infectious dose, estimated to be three bacilli (3, 49). Thus, it is crucial to understand the molecular processes that mediate the earliest stages of infection to develop vaccines that not only prevent disease but also inhibit the establishment of a successful infection.

We conducted an initial characterization of PPE19 to identify any secretion-essential relationships and assign pathogen importance. The work here demonstrates PPE19 as playing a significant role during the preliminary stages of macrophage-pathogen communication and interaction, significantly enhancing Mtb attachment and internalization. Fluorescent microspheres coated with purified PPE19 demonstrated a markedly increased rate of phagocytic uptake into macrophages, a phenotype that was replicated at the cellular level using an Mtb strain overexpressing *ppe19* (Fig. 2). Others have reported similar functions for different members of the PE/PPE protein family. It was shown that the surface-associated PPE38 is involved in *Mycobacterium marinum* entry into macrophages, with an *M. marinum ppe38* mutant displaying deficiency in macrophage invasion and a reduction in virulence compared to wild-type and complement strains (50). Another study reported that when expressed in Ms, PE_PGRS3 enhanced adhesion to human epithelial cells and murine macrophages 10-fold compared to control strains (51). The expression of PE_PGRS3 in Ms also resulted in elevated invasion into murine macrophages and enhanced colonization of the spleen in mice (51). Finally, it was shown that PE_PGRS60 binds to the extracellular matrix component fibronectin, enhancing adhesion and invasion into host cells (52). Taken together, these findings, along with the data presented in this study, support the hypothesis that PE/PPE proteins, including PPE19, may contribute to mycobacterial host-pathogen interactions by facilitating adhesion to host cells and tissues, enabling subsequent niche-specific cellular interactions, and serving to establish effective, chronic Mtb colonization.

To complement analysis of this infection-related phenotype, the expression profile of *ppe19* was evaluated through multiple means, including the use of a fluorescent dual reporter system, revealing that *ppe19* is significantly and consistently downregulated upon internalization of the bacteria (Fig. 3). The upstream promoter region of *ppe19* was transcriptionally linked to *mCherry* expression in a constitutively expressed GFP background. Flow cytometric and confocal analyses revealed a striking downregulation of *ppe19* in internalized bacteria when compared to Mtb in culture media (Fig. 3). Similarly, qRT-PCR confirmed reduced expression of ppe19 in acidified media at pH 5.4, when compared to bacteria grown at pH 7.0 (Fig. 3). This downregulation suggests that Mtb employs PPE19 as a mechanism for host cell adhesion and entry, and once internalized, no longer requires the protein as it prepares to establish persistence. Identification and understanding the function of such early antigens will contribute to vaccine development as teaching the immune system to recognize antigens crucial for establishing infection may lead to the development of vaccines not only capable of preventing disease but also able to prevent initial infection.

Mtb employs numerous secreted effector proteins, including members of the PE/PPE protein family, to interact with surface-exposed host targets and modulate immune responses during early infection. Members of the PE/PPE protein family are known to interact with an array of surface receptors to benefit infection, notably TLR2 and TLR4 (15, 53–56). PPE18 is one such protein known to interact with TLR2 to inhibit proinflammatory immune responses (31) and is required for full virulence *in vivo* (30). Furthermore, PPE18 is one of the two antigens in the M72/AS01_E_ subunit vaccine, which has demonstrated 54% protection against active pulmonary TB in a Phase 2b trial (29) and is currently undergoing Phase 3 clinical trials. PPE60 similarly stimulates the immune system through TLR-2 but drives Th1/Th17 responses (33), also making it favorable for inclusion in subunit vaccines (53). Through phylogenetic analysis, we have reported here that PPE19 falls into a distinct and exceptionally closely related clade, with PPE18 and PPE60 (Fig. 1). Although not explored in this study, this presents a hypothesis that PPE19 may signal through TLR-2, similarly to the closely related PPE18 and PPE60, to mediate macrophage phagocytosis of Mtb, a well-characterized host response to bacterial infection (57–60). Thereby, Mtb may utilize this pathway to enhance invasion into macrophages and establish infection in its intracellular niche.

The effect of overexpression or genetic knockout of *ppe19* in Mtb on virulence in a murine model was investigated, with results suggesting that the pathogen possesses functional redundancy by which it compensates for *ppe19* expression changes (Fig. 4). CRISPRi-mediated knockdown of *ppe18* and *ppe60* revealed an additive reduction in the ability of Mtb to invade host macrophages (Fig. 5). This *ppe* knockdown strain H37RvΔppe19-ppeKD was generated in the H37RvΔppe19 background due to the inability to target all three *ppe* genes with unique single guide RNAs, a limitation of the CRISPRi knockdown system when targeting highly homologous genes. This increased attenuation, combined with the similarities in the expression profiles of *ppe18*, *ppe19*, and *ppe60*, suggests that PPE18 and PPE60 may be able to compensate for PPE19. This may be due to overexpression of *ppe19,* likely increasing the rate of Mtb internalization into macrophages but not determining outright whether phagocytosis occurs. Thus, the measurement of Mtb CFUs from homogenization of the entire lung, after delivery of nearly identical inocula across both groups, is not sensitive enough to identify an alteration in virulence due to PPE19, as it does not distinguish phagocytosed bacteria from extracellular airway populations.

Through the tandem use of a mycobacterial protein fragment complementation assay and Homogeneous Time Resolved Fluorescence, we identified PE13 as an interacting partner with PPE19, presumably for its effective secretion and Mtb cell wall localization. In the complementation assay, PPE19 was fused to one domain of the murine dihydrofolate reductase enzyme, while candidate PE partners were fused to the other domain (47). These fusion proteins were produced in Ms, and resistance to trimethoprim is conferred upon PE/PPE protein interaction, allowing investigation of protein interaction in a more native mycobacterial environment (47). Previous work has identified and solved structures of a number of PE/PPE partner pairs, which has led to further understanding of their interactions and roles during infection (26, 27). This is exemplified in the well-characterized PE25/PPE41 complex (27), wherein solving their interaction has led to breakthroughs in understanding the molecular basis of PE/PPE specific secretion through ESX-5 (28, 61, 62), their role in pathogenesis and disease progression (19), and the immune recognition and response to such protein complexes (63).

Identifying PE secretion partners for PPE proteins, as we have here for PE13/PPE19, is an important first step in understanding PE/PPE structure and location and will provide new insights into defining PPE function and assigning pathogenic importance to these complex proteins. We have described the role of PPE19 in facilitating adherence and internalization into host macrophages and characterized its expression profile, highlighting the role of PPE19 in the initial stages of infection and revealing its potential for the inclusion in the development of novel subunit vaccines.

## MATERIALS AND METHODS

### Bacterial and cell culture growth conditions

*M. tuberculosis* (strain H37Rv) and *M. smegmatis* (strain mc^2^155) were cultured at 37°C in Middlebrook 7H9 broth (Difco) supplemented with 0.2% (vol/vol) glycerol, 0.05% (vol/vol) tyloxapol, and 10% (vol/vol) ADC enrichment or on Middlebrook 7H10 agar (Difco) supplemented with 0.5% (vol/vol) glycerol and 10% (vol/vol) OADC enrichment. Media contained hygromycin B (50 µg/mL) or kanamycin (25 µg/mL) as required. Anhydrotetracycline (200 ng/mL) was used to induce CRISPRi. *E. coli* DH5α or *E. coli* BL21(DE3) were cultured in Luria-Bertani (LB) broth (Difco) or on LB medium with 15 g/L agar. Media contained ampicillin (100 µg/mL), chloramphenicol (50 µg/mL), hygromycin B (100 µg/mL), or kanamycin (50 µg/mL) as required. RAW264.7 cells were grown in DMEM (Merck) and THP-1 cells in RPMI-1640 (Merck), supplemented with 10% (vol/vol) heat-inactivated fetal bovine serum (Scientifix). THP-1 monocytes were differentiated using phorbol-12-myristate-13-acetate (50 ng/mL) for 72 hours and rested for 24 hours before use.

### PPE/PE phylogeny

The amino acid sequences of PPE proteins were aligned using Muscle (version 5.1) (PMID: 15034147) with default parameters. Phylogenetic analysis was performed using IQ-TREE (version 2.0.3) ( PMID: 32011700). The best-fit substitution model, PMB + F + G4, was determined automatically using ModelFinder (PMID: 28481363) as implemented in IQ-TREE. Phylogenetic tree inference was conducted under the selected model, with branch support estimated to be using 1,000 bootstrap replicates. The resulting phylogenetic tree was visualized using the ggtree package (version 3.12.0) in R.

### Molecular techniques

Reagents used were analytical grade (Sigma-Aldrich). Recombinant plasmids were created using the homologous alignment cloning method (64). Oligonucleotide primers used were synthesized by IDT (Fig. S6). Successful construction of recombinant plasmids was confirmed by sequencing, performed at the Australian Genomics Research Centre (UQ). Transformation of mycobacteria and generation of the MtbΔ*ppe19* knockout mutant were performed as described (65).

### Mycobacterial protein fragment complementation

Ms clones containing interacting plasmids were cultured to the mid-log growth phase (OD_600_ 0.6–0.8). Cells were centrifuged (120 × *g*) to remove clumps and diluted to an OD_600_ of 0.002 in 7H9 containing trimethoprim (6.25 µg/mL). Prepared suspensions were added to 96-well microtiter plates and incubated at 37°C, with OD_600_ readings taken every 2 hours in a BMG Labtech FluoStar Omega.

### Protein expression

Overnight cultures of *E. coli* BL21(DE3) were diluted 1:20 into Terrific broth. Expression cultures were incubated at 37°C until OD_600_ 1.5–1.8. For PPE19-GST and PE13-His, anhydrotetracycline (20 ng/mL) was then added to induce pG-Tf2 chaperone expression (Takara Bio) before cultures were cooled (4°C and 20 min). IPTG (0.5 mM) induced protein expression in all cultures before overnight incubation at 15°C for PPE19-GST and PE13-His, or 37°C for PPE19. Cells were harvested by centrifugation (4,500 × *g*).

### PPE19-GST purification

Expression cell pellets were resuspended in lysis buffer: 50 mM Tris-HCl, pH 7.4, 20 mM NaCl, 10 mM MgCl_2_, 50 µg/mL DNase I (Sigma), 100 µg/mL lysozyme (Sigma), 1 mM PMSF, and “cOmplete” EDTA-free protease inhibitor (Roche). Suspensions were sonicated, and then lysates were clarified by centrifugation (45,000 × *g*) before passage through a 0.45 µm PVDF filter. Clarified lysates were injected onto a GSTrap 4B column (1 mL) (Cytiva) using an AKTApure fast protein liquid chromatography (FPLC) system. The column was extensively washed with buffer A (50 mM Tris-HCl, pH 7.4, and 20 mM NaCl) before elution with buffer B (50 mM Tris-HCl, pH 8.5, 150 mM NaCl, 45 mM reduced glutathione, and 0.1% [wt/vol] Triton X-100). Elution fractions containing PPE19-GST were identified by SDS-PAGE, pooled, and dialyzed into buffer C (50 mM HEPES, pH 7.4, 150 mM NaCl, and 5% [vol/vol] glycerol). The subsequent sample was concentrated using a centrifugal filter (10 kDa cut off, Amicon) before injection into a HiPrep Sephacryl S-200 HR (size-exclusion) column (26/60) (Cytiva), which was washed extensively with buffer C. Elution fractions containing PPE19-GST were collected, concentrated using a centrifugal filter, snap-frozen in liquid nitrogen, and stored at −80°C. When required, PPE19-GST was biotinylated using the EZ-Link Sulfo-NHS-LC-Biotinylation Kit (ThermoFisher Scientific), with passage through a Zeba Spin Desalting Column (ThermoFisher Scientific) to remove unbound biotin, according to the manufacturer’s instructions.

### PE13 purification

Expression cell pellets were lysed and clarified as described for PPE19-GST, except that the lysis solution was HEPES-buffered. PE13 was purified using TALON Metal Affinity resin (TakaraBio), following the batch gravity method described by the manufacturer, with wash buffer (50 mM HEPES, pH 7.4, 150 mM NaCl, and 5 mM imidazole) and elution buffer (50 mM HEPES, pH 7.4, 150 mM NaCl, and 300 mM imidazole) optimized for PE13-His purification in this study.

### HTRF

A volume of 5 µL of PPE19-GST-bio and 5 µL of PE13-His, diluted in “HTRF buffer” (50 mM HEPES, pH 7.4, and 150 mM NaCl), was added to the relevant wells of a Grenier 384-well black-wall plate on ice, to the indicated concentrations. The reaction was incubated for 30 min at 4°C, before addition of 10 µL of combined Cisbio HTRF detection reagents (terbium cryptate anti-6His and streptavidin-d2), previously reconstituted in “HTRF buffer” containing 0.1% (wt/vol) BSA, 0.1% (vol/vol) Triton X-100. After 1 hour of incubation at 4°C, HTRF signals were measured using a CLARIOstar plate reader, with measurement conditions as described by Cisbio. HTRF signals were computed ratiometrically. Briefly, specific signals for each reaction were calculated by dividing the 665 nm signal by the 620 nm signal, before multiplication by 104 and subtraction of the “background sample” ratio. To normalize across replicate experiments, Δ*F* values were obtained for each well by dividing the calculated specific signal by the “background sample” value. Within each experiment, these Δ*F* values were then divided by the highest ΔF value within that experiment to obtain Δ*F*/Δ*F*-max values. Calculated Δ*F*/Δ*F*-max values indicate unbiased, ratiometric protein-protein interaction values corrected for background signal and variability across experiments, with an internal control of fluorescence emission at 620 nm.

### PPE19 purification

*E. coli* BL21(DE3), containing pET19b-ppe19, was used to express PPE19 inclusion bodies. PPE19 expression, purification, and refolding were performed essentially as described (66). Prior to use, samples were dialyzed into PBS and treated with a Pierce high-capacity 0.5 mL endotoxin removal spin column (ThermoFisher Scientific), according to the manufacturer’s instructions. Successful removal of endotoxin was quantified using an assay of ELAM promoter activity, essentially as described (34), with LPS from *E. coli* 026:B6 as a positive control (ThermoFisher Scientific).

### Microsphere uptake assay

FluoSpheres Sulfate Microspheres (1.0 µm, red fluorescent [580/605]) (Invitrogen) were coated by passive adsorption (24 hours at 4°C) with PPE19 or BSA at 50 µg/mL in PBS. Successful passive adsorption was quantified by bicinchoninic acid assay before all beads were briefly backcoated with high concentration BSA (~1 mg/mL) to bind remaining hydrophobic areas. RAW264.7 cells (67) were seeded onto sterile coverslips to reach 1.5 × 10^6^ cells/well. PPE19-coated or BSA-coated beads were applied to the cells to achieve an MOI of 4. At 4 hours post-infection, monolayers were washed thrice with PBS. Monolayers were then fixed with paraformaldehyde (4% [wt/vol]) at room temperature (30 min). Macrophages were stained with wheat-germ agglutinin Alexa Fluor 488 conjugate (WGA-488) and NucBlue Fixed Cell Reagent (ThermoFisher Scientific). Coverslips were mounted onto microscope slides using Dako fluorescence mounting medium. Samples were imaged using an LSM 900 Confocal microscope (Zeiss), and macrophage and microsphere numbers were quantified using ImageJ.

### Mtb infection assays

THP-1 cells were differentiated at 2 × 10^5^ cells/well. Mtb strains were grown to the mid-log phase (OD_600_ 0.6–0.8), pelleted, and resuspended in RPMI-1640. Single-cell suspensions were obtained by de-clumping through vortexing with 1 mm glass beads, followed by passage through a 5 µm PVDF filter and dilution in media to achieve the desired MOI. Regardless of the total infection length, monolayers were washed thrice with warm PBS after 4 hours. To analyze internalization, infected monolayers were pulsed with amikacin (200 µg/mL, 45 min) after washing to kill extracellularly adhered bacteria. After the pulse treatment, the monolayers were washed three additional times before being lysed with Triton X-100 (0.1% [vol/vol] in PBS). Lysates were serially diluted before plating on 7H10 agar. Mtb CFUs were counted after 18–21 days of incubation at 37°C. For adherence analysis, infected macrophages were lysed prior to amikacin treatment and plated as described. Approximate adhered CFUs were then calculated by subtraction of the mean internalized CFUs from these counts. For longer infections, infected monolayers were washed and amikacin was pulsed as above. Monolayers were then maintained at 37°C and 5% CO_2_ for 5 days, with culture medium replaced every 48 hours. At indicated time points, monolayer lysis and bacterial plating were performed as described. Macrophage integrity was monitored daily using an inverted light microscope (Leica Dmi1).

### RNA extraction and qRT-PCR

Mtb strains were grown to the mid-log growth phase and exposed to the condition of interest, if necessary, before centrifugation and stabilization of RNA transcripts using RNAprotect Bacteria Reagent (Qiagen). Protected pellets were resuspended in Buffer RLT (Qiagen) containing β-mercaptoethanol (1% [vol/vol]) and disrupted by bead-beating using 0.1 mm zirconia beads before resultant lysates were mixed with chloroform. Following centrifugation, the resultant aqueous phase was collected, and total RNA was subsequently extracted using the RNeasy mini kit (Qiagen). Contaminating genomic DNA was digested with RNase-free DNase I (Ambion) prior to ethanol precipitation to re-purify RNA. cDNA was prepared from 1 µg of DNA-free total RNA using the Tetro cDNA synthesis kit (Bioline) with random hexamers. cDNA levels were then measured by qRT-PCR on a QuantStudio6 (ThermoFisher Scientific) using QuantiFast SYBR Green PCR mix (Qiagen). All primer pairs were verified to be >90% efficient for their target. Data analysis was performed using the delta-delta Ct method (68), with signals normalized to the housekeeping *rpoB* transcript.

### pDual-Pppe19 reporter infections

THP-1 cells were differentiated at 2 × 10^6^ cells/well and then infected with a single-cell suspension of H37Rv::pDual-P*ppe19*. For flow cytometry analysis, monolayers were washed with PBS 4 hours post-infection and lysed with Triton X-100 (0.1% [vol/vol]). Mtb was harvested from the lysate by centrifugation and immediately fixed with paraformaldehyde (4% [wt/vol]). Following fixation, samples were pelleted and resuspended in PBS-Tyloxapol (0.05% [vol/vol]). Mtb was identified within lysates by GFP fluorescence, and the median mCherry fluorescence of this gated population was recorded as a measure of *ppe19* promoter activity (~10,000 events per sample). H37Rv::pDual-P*ppe19,* fixed directly from culture, provided a baseline measure of *ppe19* promoter activity. For samples to be analyzed by microscopy, the infection proceeded as above, except THP-1 cells were fixed directly on sterile coverslips. Macrophages were then stained with wheat-germ agglutinin Alexa Fluor 647 conjugate and NucBlue Fixed Cell Reagent (ThermoFisher Scientific). Coverslips were mounted onto microscope slides using Dako fluorescence mounting medium. Samples were imaged using an LSM 900 Confocal microscope (Zeiss) and analyzed as previously described (67). H37Rv::pDual-P*ppe19* fixed directly from culture and dried onto microscope slides was used as a baseline measure of *ppe19* promoter activity.

### Mouse infections

Mice were sourced from the Animal Resources Centre (Perth, Australia). Animals were contained within a Techniplast housing system inside of physical containment level 3 facility, with food and water supplied *ad libitum*. Mtb strains were grown to the mid-log phase and then prepared as single-cell suspensions in PBS. Female 6–8-week-old C57BL/6 mice were infected by aerosol with between 10^2^ and 10^3^ CFU, using an Inhalation Exposure System (Glas-Col). Inocula were confirmed through plating on 7H10 agar and incubation at 37°C for 21 days. At indicated time points, mice were euthanized through CO_2_ asphyxiation. Bacterial burdens were determined through the dissection, homogenization, and plating of desired organs. Serial dilutions of each organ homogenate were plated onto 7H10 agar, and bacterial counts were enumerated after 21–25 days of incubation at 37°C.

### Statistical analysis

GraphPad Prism was utilized to prepare graphs and for statistical analysis. All data are presented as mean with standard deviation. Significance was calculated using analysis of variance with Tukey’s or Sidak’s multiple comparisons test or unpaired *t*-test. Significance was considered at *P* < 0.05.

## SUPPLEMENTAL MATERIAL

Supplemental File msphere.00036-25-s0001.docx Supplemental figures and tables.

ASM does not own the copyrights to Supplemental Material that may be linked to, or accessed through, an article. The authors have granted ASM a non-exclusive, world-wide license to publish the Supplemental Material files. Please contact the corresponding author directly for reuse.
