# Supplementary material for: Characterization of PPE19 as a novel mediator of Mycobacterium tuberculosis-macrophage interactions
Source: mSphere. 2025 Aug 11;10(9):e00036-25. doi: 10.1128/msphere.00036-25 (PMC12482190; doi:10.1128/msphere.00036-25)
Supplement: Supplemental File — Supplemental figures and tables. [file msphere.00036-25-s0001.docx]

Supplementary Material

S1A – Phagocytosis of fluorescent microspheres coated with R-PPE19


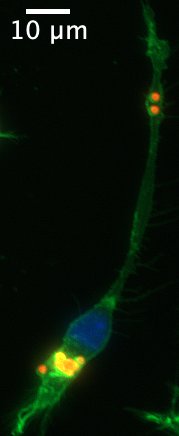

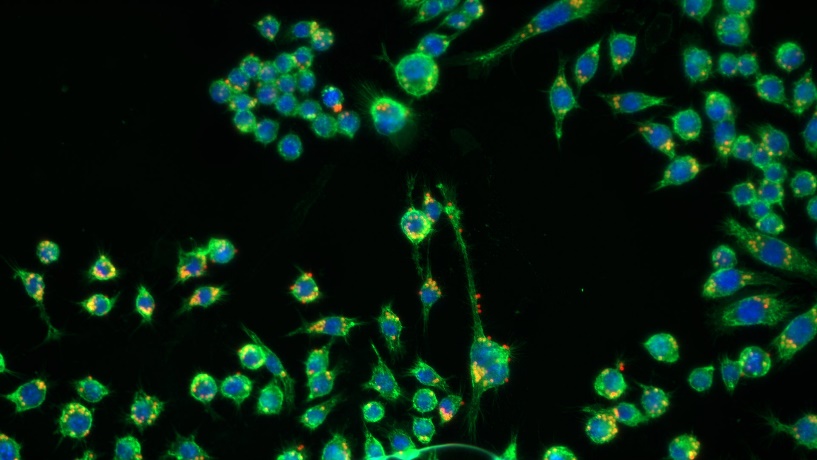


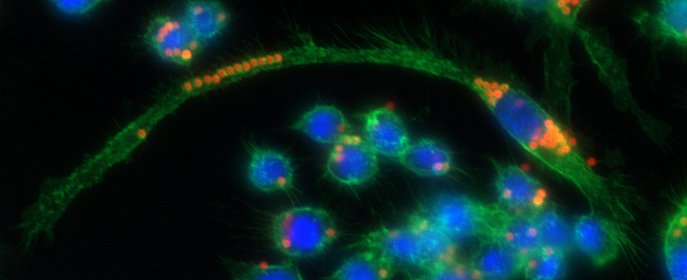


**FIG S1A** Fluorescent microspheres coated with R-PPE19 are phagocytosed by macrophages. FluoSpheresTM Sulfate Microspheres (1.0 μm, red fluorescent (580/605)) (Invitrogen), were coated by passive adsorption (24 hours, 4℃) with PPE19 or BSA at 50 μg/mL in PBS. Coated microspheres were applied to RAW264.7 macrophages at MOI 4. Macrophages were stained with wheat-germ agglutinin Alexa Fluor 488 conjugate (WGA-488) and NucBlue Fixed Cell Reagent (ThermoFisher Scientific) and imaged using a LSM 900 Confocal microscope (Ziess).

S1B – Purified R-PPE19


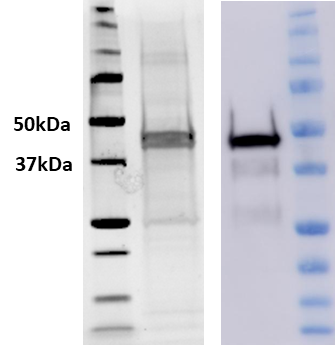


**FIG S1B** Purification of R-PPE19. SDS-PAGE and Western blot of R-PPE19 following purification by immobilised metal affinity chromatography. Western blot performed using an anti-polyhistidine primary antibody (Invitrogen, 1:2,000), anti-mouse IgG secondary antibody (Invitrogen, 1:20,000), and visualised by chemiluminescence.

S1C – Endotoxin quantification in R-PPE19


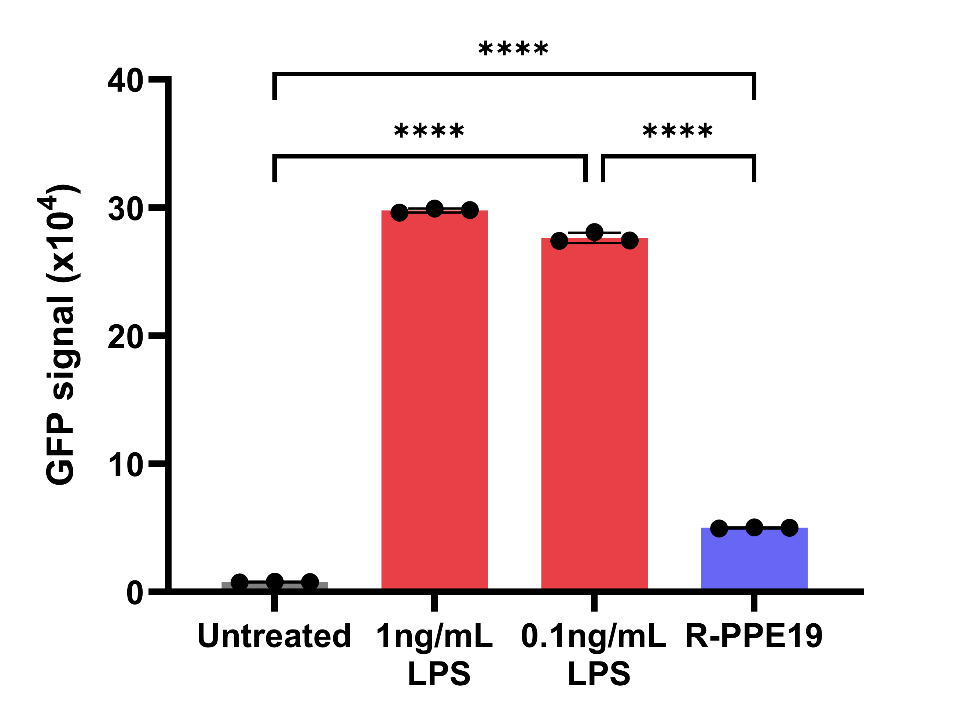


**FIG S1C** RAW264.7-ELAM assay demonstrating TLR activation through GFP fluorescence, following treatment with LPS (1 or 0.1 ng/mL) or R-PPE19 (1 μg/mL). Graph is representative of duplicate experiments and data points are technical replicates of median GFP signal from 20,000 events.

S2 – THP-1 extended infection


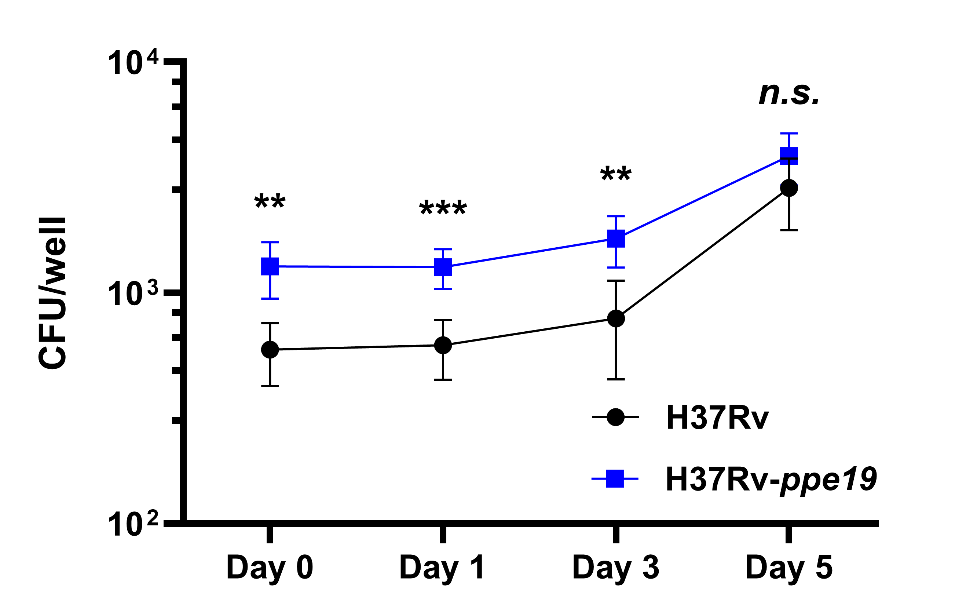


**FIG S2** Burden of H37Rv or H37Rv-*ppe19* in THP-1 macrophages over 5-day infection at MOI of 0.1. Graph is representative of 3 biological replicates and data points indicate 6 technical replicates. Data are expressed as mean with SD. Significance was calculated using two-way ANOVA (with Sidak’s multiple comparisons test). **, p<0.01; ***, p<0.001.

S3 – Representative micrographs of pDual-*ppe19* infection


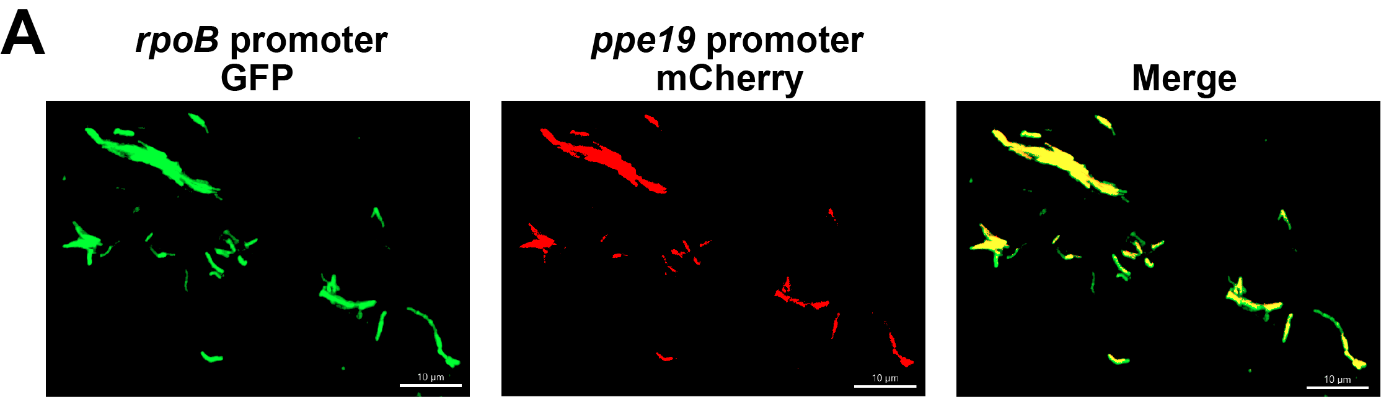


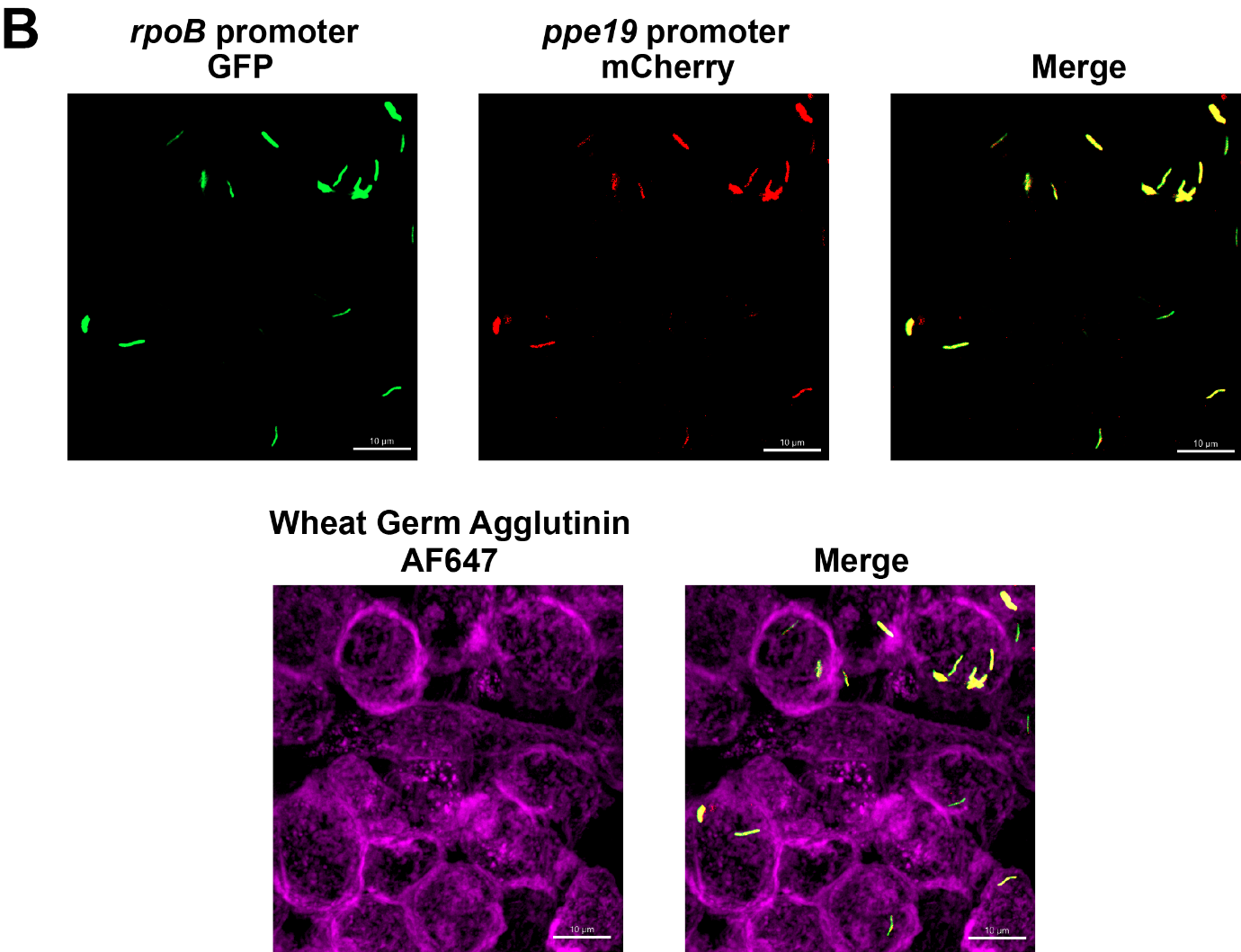


**FIG S3** Expression of *ppe19* is downregulated following Mtb infection of macrophages. Level of mCherry fluorescence was measured in H37Rv::pDual-P*ppe19* grown in 7H9 media (A) or following infection of THP-1 macrophages for 4 hours at MOI of 5 (B). Macrophages were stained with wheat-germ agglutinin Alexa Fluor 647 conjugate (Invitrogen) and samples were imaged using a LSM 900 Confocal microscope (Ziess).

S4 – Knockdown efficiency of *ppe* single guide RNAs


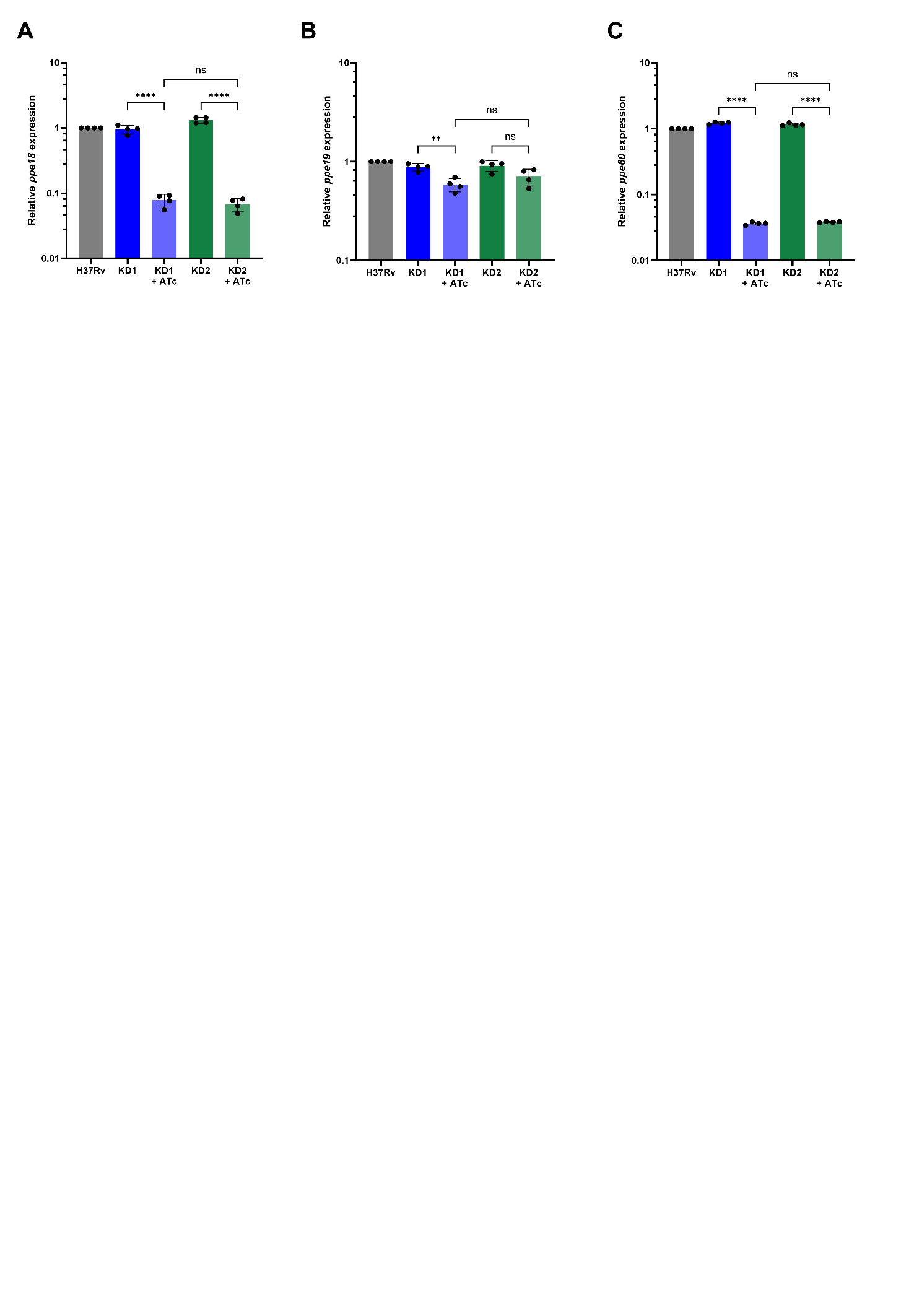


**FIG S4** qRT-PCR data of triple *ppe18,19,60* knockdown from 2 different guide RNAs designed for each gene. + Atc refers to induced knockdown, whilst ‘KD1/2’ refer to Mtb containing the vector, but no CRISPRi induction. Data are expressed as mean with SD. Significance was calculated using one-way ANOVA (with Tukey’s multiple comparisons test) **, p<0.01; ****, p<0.0001.

S5 – Purification of PPE19-GST-bio and PE13-His


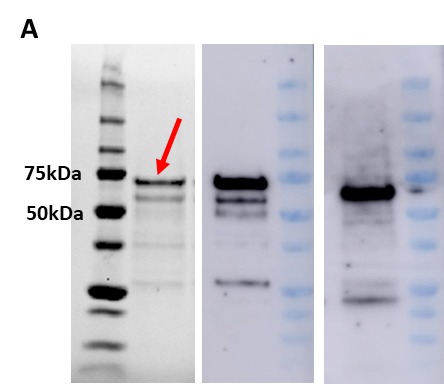

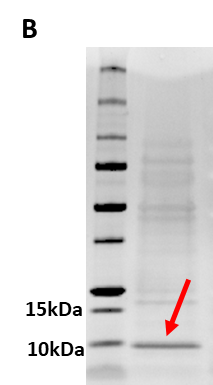


**FIG S5** Purification of PPE19-GST-bio and PE13-His. (A) SDS-PAGE, Western blot and Ligand blot of PPE19-GST-bio. SDS-PAGE and Western blot of PPE19-GST following purification by GSTrap and size-exclusion chromatography. Western blot performed using anti-GST primary antibody (Invitrogen, 1:1,000), anti-mouse-IgG secondary antibody (Invitrogen, 1:20,000) and visualised by chemiluminescence. (B) SDS-PAGE of PE13-His following purification by TALON resin.

S6 – Plasmids used in this study

| **Plasmid Name** | **Description** | **Source** | |
| --- | --- | --- | --- |
| pMV206 | Promoter-less *E. coli*-mycobacterial expression vector; MCS, OriE, OriM, Kan^R^. | Stover (1991) | |
| pMV261 | *E. coli*-mycobacterial expression vector with constitutive Hsp60 promoter; MCS, OriE, OriM, Kan^R^. | Stover (1991) | |
| pMV306 | Promoter-less *E. coli*-mycobacterial expression vector; MCS, OriE, L5 integrase, Kan^R^. | Stover (1991) | |
| pMV361 | *E. coli*-mycobacterial expression vector with constitutive Hsp60 promoter; MCS, OriE, L5 integrase, Kan^R^. | Stover (1991) | |
| **Altered *ppe19* Expression** | | | |
| pMV261-*ppe19* | pMV261 containing *ppe19* gene sequence. | This study | |
| pMV306-*ppe19* | pMV306 containing 554bp *ppe19* upstream region and *ppe19* gene sequence. | This study | |
| pMV361-*ppe19* | pMV361 containing *ppe19* gene sequence. | This study | |
| pJV53 | Recombineering vector containing Che9c mycobacteriophage recombinases; OriE, OriM, Kan^R^. | van Kessel and Hatfull (2007) | |
| pYUB854 | Mycobacterial suicide plasmid; dual MCS, OriE, Hyg^R^ flanked by *γδ-res* sites. | van Kessel and Hatfull (2007) | |
| pYUB854-*ppe19* | pYUB854 containing 1kb of both up and downstream genomic regions of the *ppe19* gene sequence, separated by Hyg^R^. | This study | |
| pYUB870 | pMV261 containing *γδ-*resolvase (*tnpR*) for removal of Hyg^R^ gene at *γδ-res* sites. | Jacobs, 2002 | |
| **Fluorescent Reporters** | | |  |
| pDual206 | pMV206 containing promoter-less *mCherry* and PrpoB-*gfp*  promoter-reporter fusion. | Stupar (2024) |  |
| pDual-P*ppe19* | pDual206 containing 554bp *ppe19* upstream region fused to *mCherry.* | This study |  |
| **CRISPRi Constructs** | | |  |
| pLJR965 | *Mtb* CRISPRi vector; MCS, OriE, L5 integrase, TetR, TetO, Kan^R^. | Rock (2017) |  |
| pLJR965-ppeKD1 | PLJR965 encoding sgRNA KD1 for simultaneous knockdown of *ppe18, ppe19,* and *ppe60.* | This study | |
| pLJR965-ppeKD2 | PLJR965 encoding sgRNA KD2 for simultaneous knockdown of *ppe18, ppe19,* and *ppe60.* | This study | |
| **Protein Expression** | | | |
| pET19b | *E. coli* vector for IPTG-inducible expression of 10xHis-tagged proteins; MCS; OriE; Amp^R^. | Novagen | |
| pET19b-*ppe19* | pET19b containing *ppe19*, codon optimised for expression in *E. coli.* | This study | |
| pET19b-GST | pET19b with 10xHis-tag excised and replaced with GST. | This study | |
| pET19b-GST-*ppe19* | pET19b-GST containing *ppe19*, codon optimised for expression in *E. coli.* | This study | |
| pG-Tf2 | *E. coli* vector for tetracycline-inducible expression of GroEL-GroES and trigger factor chaperones; OriE; Cm^R^ | Takara Bio | |
| **Two-Hybrid System** | | |  |
| pUAB300 | pMV206 containing fragments 1&2 of DHFR gene, upstream of 10xGly linker sequence and MCS. Hyg^R^ in place of Kan^R^. | Singh, 2006 |  |
| pUAB400 | pMV306 containing fragment 3 of DHFR gene, upstream of 10xGly linker sequence and MCS. | Singh, 2006 |  |
| pUAB300-pe13 | pUAB300 containing *pe13* fused to C-terminus of DHFR (1,2). | This study |  |
| pUAB300-pe25 | pUAB300 containing *pe25* fused to C-terminus of DHFR (1,2). | This study |  |
| pUAB300-pe31 | pUAB300 containing *pe31* fused to C-terminus of DHFR (1,2). | This study |  |
| pUAB400-ppe19 | pUAB400 containing *ppe19* fused to C-terminus of DHFR (3). | This study |  |
| pUAB400-ppe41 | pUAB400 containing *ppe41* fused to C-terminus of DHFR (3). | This study |  |

Abbreviations: OriE, *E. coli* origin of replication; OriM, mycobacterial origin of replication; MCS, multiple cloning site; L5 int, mycobacteriophage L5 integration cassette; Kan^R^, kanamycin resistance marker; Hyg^R^, hygromycin resistance marker; Amp^R^, ampicillin resistance marker; Cm^R^, chloramphenicol resistance marker.

S7 – Oligonucleotides used in this study

The primers used throughout this investigation. Primers incorporating homologous alignment cloning tails (64) are underlined.

| **Name** | **Sequence** |  |
| --- | --- | --- |
| **Construction of pMV261-*ppe19*** | | |
| cpm24 | AGCTGCAGAATTCGAAGCTGGAAGGATTATCGAAGTGGTG |  |
| cpm24a | CGACATCGATAAGCTTTACCCGGCGGCGGGCACACG |  |
| **Construction of pMV306-*ppe19*** | | |
| wpr1328 | GCAGAATTCGAAGCTCAGTTCACGCAGCAGATCGTGGC |  |
| wpr1330 | CGACATCGATAAGCTTTACCCGGCGGCGGGC |  |
| **Construction of pMV361-*ppe19*** | | |
| wpr1331 | ATGGCCAAGACAATTGTGGTGGACTTCGGGGCGTTAC |  |
| wpr1333 | GCTGGATCCGCAATTTTACCCGGCGGCGGGC |  |
| **Construction of pYUB854-*ppe19*** | | |
| wpr1223 | AGCTCACCTAGGTATCTAGCGTACCGTGACCGATGACG |  |
| wpr1224 | TAGGATACACCGGTTCTAGTTCGATAATCCTTCCGCTCGG |  |
| wpr1225 | ACGCGCACCATGGGAAGCTGTAACGCCGATCCGCAC |  |
| wpr1226 | ATCTGGATCCACGAAGCTCCAACATCGCGGTAGCGTTCG |  |
| **PCR confirmation of Δ*ppe19*** | | |
| wpr1229 | CGTCGAATCCATGAACGAGC |  |
| wpr1230 | CGGCGTGTCCTGCATACTGG |  |
| wpr1231 | GTTACCACCGGAGATCAACTC |  |
| wpr1232 | CCTTCAACATCGAGTGC |  |
| **Construction of pDual-P*ppe19*** | | |
| wpr807 | GATCTTTAAATCTAGCAGTTCACGCAGCAGATC |  |
| wpr808 | TTCTCCTTCTGATCTAGTTCGATAATCCTTCCGCTCG |  |
| **Construction of pLJR65-ppeKD1** | | |
| wpr1420 | CAGTGATAGATATAATCTGGGAGCCTCCTCGACCGCGACGGCCTGCT |  |
| wpr1421 | TCTTTCGAGTACAAAAACAGCAGGCCGTCGCGGTCGAGGAGGC |  |
| **Construction of pLJR65-ppeKD2** | | |
| wpr1422 | CAGTGATAGATATAATCTGGGACCCATACGCCGTCTCGTAGGCCGCC |  |
| wpr1423 | TCTTTCGAGTACAAAAACGGCGGCCTACGAGACGGCGTATGGG |  |
| **Construction of pET19b-*ppe19*** | | |
| wpr1007 | GACGACAAGCATATGGTGGTTGACTTTGGTGCGCT |  |
| wpr1008 | ATCCTCGAGCATATGTTAACCCGCCGCCGGAACAC |  |
| **Construction of pET19b-GST** | | |
| wpr687 | CCGGATCCTCGAGCATAGGATTGGAAGTACAGGTTC |  |
| wpr658 | CCGGATCCTCGAGCATAGCCCTGAAAATAAAGATTCTCtGTACCCAGATCTGGGTAATC |  |
| **Construction of pET19b-GST-*ppe19*** | | |
| wpr1098 | CAATCCTATGCTCGAGTGGTTGACTTTGGTGCGCT |  |
| wpr1100 | CGGATCCTCGATTAACCCGCCGCCGGAACAC |  |
| **Construction of pUAB300-*pe13*** | | |
| wpr1179 | ATTCGAAGCTTATCGGTGTCTTTCGTGATGGCA |  |
| wpr1180 | ACTACGTCGACATCGTTCTCGTGCCGACTGTTC |  |
| **Construction of pUAB300-*pe25*** | | |
| wpr1177 | ATTCGAAGCTTATCGATGTCTTTTGTGATCACAAATC |  |
| wpr1178 | ACTACGTCGACATCGTTAACTAAAGGTCTTGATGTTG |  |
| **Construction of pUAB300-*pe31*** | | |
| wpr1181 | ATTCGAAGCTTATCGGTGTCTTTCACTGCGCAA |  |
| wpr1182 | ACTACGTCGACATCGTCCGCTCGAATACCGTC |  |
| **Construction of pUAB400-*ppe19*** | | |
| wpr1175 | TGGTGGGTCCCAATTGGTGGTTGACTTTGGTGCGCT |  |
| wpr1176 | ACTACGTCGACATCGTTAACCCGCCGCCGGAACAC |  |
| **Construction of pUAB400-*ppe41*** | | |
| wpr1173 | TGGTGGGTCCCAATTGATGCATTTCGAAGCGTA |  |
| wpr1174 | ACTACGTCGACATCGCTAAGTGTCTGTACGCGA |  |
|  |  |  |
| **RT-qPCR primers** | | |
| ***rpoB*** | | |
| wpr884 | TCGTTTCGACGATGTCAAGG |  |
| wpr885 | GTCATCATCGGGAAGTCACC |  |
| ***ppe18*** | | |
| wpr1398 | ACCAGCGCCGCGGAAAGAG |  |
| wpr1399 | ATGCGGCATCACATAGGGTCGC |  |
| ***ppe19*** | | |
| wpr918 | TGAACTCTGGAAAGCCATCTC |  |
| wpr919 | GAGCAAAGCCCTTCAACATC |  |
| ***ppe60*** | | |
| wpr1400 | GCGTCGTACCTTCTTCCAAGC |  |
| wpr1401 | GTCATCGACACACCCGTGC |  |
